# Histopronostic factors in superficial colorectal adenocarcinomas treated by endoscopy: reproducibility and impact of immunohistochemistry and digital pathology

**DOI:** 10.1007/s00428-023-03722-3

**Published:** 2024-01-26

**Authors:** Guillaume Pontarollo, Maxime Bonjour, Thomas Walter, Mathieu Pioche, Pierre-Marie Lavrut, Maud Rabeyrin, Valérie Hervieu, Tanguy Fenouil

**Affiliations:** 1 Hospices Civils de Lyon, Institut de Pathologie Multi-sites, site EST, Groupement Hospitalier Est, 59 boulevard Pinel, 69677 Bron, France; 2 Université de Lyon, Université Lyon 1, Lyon, France; 3 Service de Biostatistique-Bioinformatique, Pôle Santé Publique, Hospices Civils de Lyon, Lyon, France; 4 Hospices Civils de Lyon, service d’oncologie médicale, Hôpital Édouard Herriot, Lyon, France

**Keywords:** Colorectal cancer, Reproducibility, Prognosis, Endoscopic sample, Pathologic examination

## Abstract

Endoscopic dissection is the first-choice treatment for superficial pT1 colorectal adenocarcinoma (sCRC). Complementary surgery decision is influenced by histopronostic factors. Prognostic significance and reproducibility of each factor are not well established. The role of immunohistochemistry (IHC) and digital pathology in this context is unknown. Our aims were (1) to evaluate each histopronostic factor reproducibility comparing HES and IHC ± digital pathology and (2) to evaluate how the different techniques would affect indications for additional surgery. We performed a single-centre retrospective study of 98 patients treated between 2010 and 2019 in Hospices Civils de Lyon, France. We analyzed physical or digital slides of HES and keratin/desmin immunostaining of 98 sCRC dissection specimens. Three pathologists evaluate the histopronostic factors including submucosal invasion depth (SMI) measured using different recommended methods. Assessment of SMI with Ueno or JSCCR methods showed good to excellent interobserver reproducibility (IOR) (ICCs of 0.858 to 0.925) using HES staining and IHC. Assessment of budding on HES sections was poorly reproducible compared to IHC which exhibit moderate IOR (*κ* = 0.714). IHC increased high-grade budding detection. For lymphovascular invasion and poor differentiation, the IOR was poor (*κ* = 0.141, 0.196 and 0.313 respectively). IHC gave a better reproducibility for further treatment indication according to JSCCR criteria (*κ* = 0.763) or forthcoming European guidelines (*κ* = 0.659). Digital pathology was equivalent to the microscope for all analyses. Histopronostic factor reproducibility in sCRC is moderate. Immunohistochemistry may facilitate the evaluation of certain criteria and improve the reproducibility of treatment decisions.

**Supplementary Information:**

The online version contains supplementary material available at 10.1007/s00428-023-03722-3.

## Introduction

Colorectal adenocarcinoma (CRC) is the second most common cancer in women and the third most common in men with an estimated 1,849,418 new cases worldwide in 2018 [1]. More colorectal cancers are now diagnosed at an early stage thanks to advances in screening, and digestive endoscopy techniques allow an increasing number of early-stage cancers to be removed. Endoscopic treatment is also less invasive and has lower morbidity compared with traditional surgery [2].

Lymph node metastases are found in between 3.6 and 16.2% of patients with superficial pT1 colorectal cancers (sCCR), conditioning their eligibility for endoscopic treatment alone or for additional surgery with lymph node dissection [3]. According to current international guidelines, including those of the Japanese Society for Cancer of the Colon and Rectum (JSCCR), incomplete resection, significant budding (grade 2 or 3), venous and/or lymphatic invasion, adenocarcinoma with poor differentiation and submucosal invasion (SMI) deeper than 1000 μm are indications for surgery [4–6]. Whether forthcoming European guidelines will endorse the same indications or will propose another SMI threshold of 2000 μm remains uncertain. Indeed, some studies suggest that in the absence of other indications, a SMI threshold > 1000 μm, alone, is not associated with higher risk of lymph node metastases or poorer survival [7–12]. These parameters are widely accepted but they suffer from variable interobserver agreement [13–20]. Moreover, there are some debate in the literature about how best to measure SMI depth and what threshold, ranging from 1000 to 3000 μm, best predicts the risk of lymph node metastasis [4, 7, 9, 21, 22]. Three quantitative methods have been proposed since the turn of the century and the corresponding measurement differences can affect patient management [4, 7, 9]. Interobserver agreement has only ever been assessed for the Ueno method, and interobserver and intermethod variability, with or without immunohistochemistry (IHC) and/or digitized slides, is an important concern [13–15]. The use of IHC, whether to measure infiltration or to assess budding, is not yet well established, and while digital pathology is increasingly used for diagnosis, its place in the evaluation of these criteria has not been studied.

The aims of this study were therefore to evaluate (i) the reproducibility of histopronostic factors to guide patient management after endoscopic resection of superficial colorectal cancer and (ii) the contributions of IHC and digital pathology in evaluating these criteria and the impact of these techniques on indications for additional surgery in current international guidelines and forthcoming European recommendations.

## Methods

### Patients

All patients who had a pT1 sCCR treated by endoscopic resection between 01/01/2010 and 31/12/2019 in the study centre (department of gastroenterology, Edouard Herriot Hospital, Lyon France) were included. Patients were identified exhaustively by cross-referencing database of the sample management software (Diamic, Dedalus C&G) from our pathology department with patient lists from multidisciplinary gastrointestinal tumour board (MDT). The exclusion criteria were insufficient material for immunohistochemical study, no visible infiltrating cells left on immunohistochemical slides and the tumour being reclassified to a higher stage than pT1 on examination of the additional surgical specimen. Clinical data on follow-up, overall survival, metastasis-free survival and recurrence were collected from the patient’s medical record.

### Endoscopic data

The endoscopic data considered were the location of the tumour, its size and the type of resection (endoscopic submucosal dissection (ESD), endoscopic mucosal resection (EMR), endoscopic piecemeal mucosal resection (EPMR)), as recorded in patient’s endoscopy reports.

### Sample processing and immunochemistry

The analyzed slides were 4 μm haematoxylin-eosin-saffron (HES)-stained tissue sections. Dual colour IHC was performed using a Ventana BenchMark ULTRA® automated slide preparation system (Ventana-Roche Diagnostics), an UltraView DAB Detection Kit (Ventana-Roche Diagnostics) and an UltraView Alkaline Phosphatase Red Detection Kit (Ventana-Roche Diagnostics) with the following antibodies: AE1/AE3 keratin (1:400 dilution, Dako); D33 desmin (1:50 dilution, Dako).

### Measurement of histological parameters

All HES slides with infiltrating cells were independently analyzed by three pathologists (GP, TF and VH), respectively a junior, a senior and a senior pathologist specialized in gastrointestinal pathology. The HES and IHC slides were digitized with a Leica biosystems Aperio AT2 brightfield scanner. The parameters only evaluated on physical slides were the type of polyp, lymphovascular invasion (lymphatic and venous invasion was differentiated based on the absence/presence of muscular layer in the invaded vessel), histological grade according to the 2010 World Health Organization classification and the 2019 WHO classification, mucinous or signet ring cells in the deepest part of tumour and presence of a positive vertical margin as recommended by the JSCCR [4, 23]. Whenever possible, the SM level of invasion was classified according to Kikuchi et al. [24].

Tumour buds were counted according to the recommendations of the 2016 International Tumour Budding Consensus Conference (single cells or clusters of < 5 cancer cells without gland formation at the front of the tumour/0.785 mm^2^) [5]. Tumour budding was then scored in a three-tiered (grade 1 to grade 3) and two-tiered system (not significant: grade 1 or significant: grade 2 and 3).

**Fig. 1:**
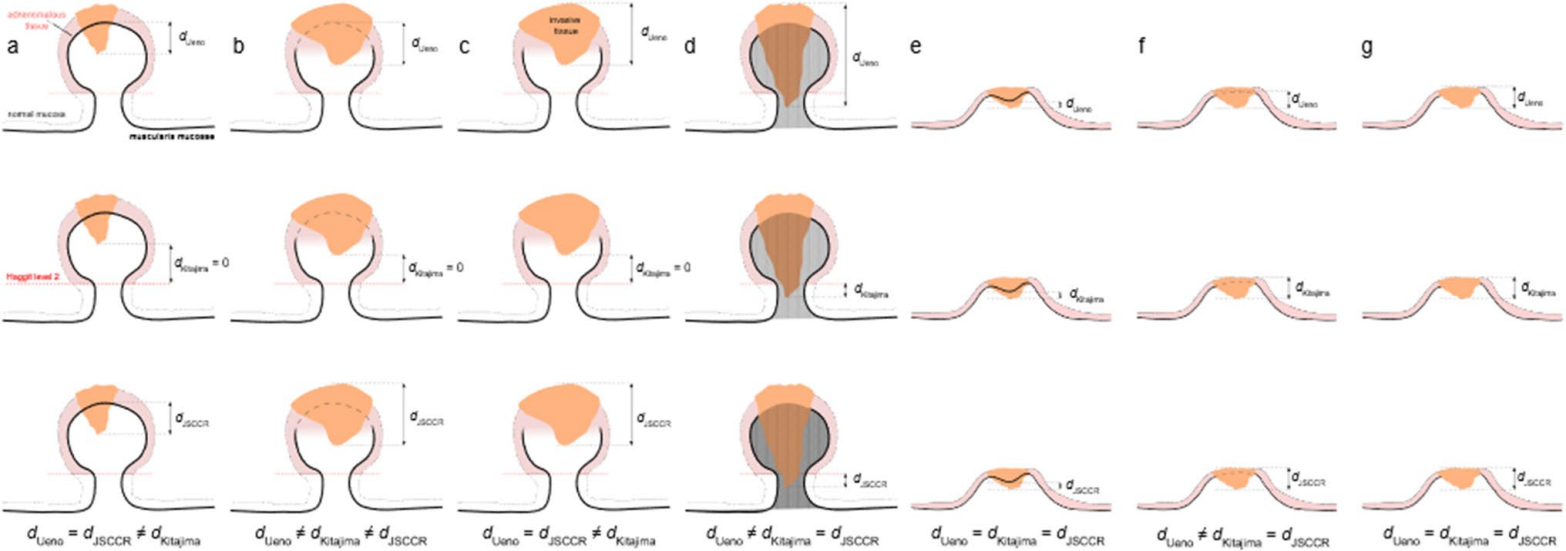
Measurement of submucosal invasion depth using the methods proposed by Ueno et al. [9] (top row), Kitajima et al. [7] (middle row) and the Japanese Society for Cancer of the Colon and Rectum [4] (JSCCR, bottom row), for different forms of invasion in pedunculated and sessile polyps: (a) a pedunculated polyp with head invasion and a visible, intact muscularis mucosae; (b) a pedunculated polyp with head invasion and a damaged but locatable muscularis mucosae; (c) a pedunculated polyp with head invasion and an invisible or altered muscularis mucosae; (d) a pedunculated polyp with head and stalk invasion and a tangled muscularis mucosae; (e) a sessile polyp with a visible, intact muscularis mucosae; (f) a sessile polyp with a damaged but locatable muscularis mucosae and (g) a sessile polyp with an invisible or altered muscularis mucosae

The depth of SMI that was measured in micrometres according to the Ueno, Kitajima and JSCCR methods (Fig. 1) [9, 7, 4]. The measurements were made either with an optical micrometre under microscope or using the Aperio ImageScope software (Leica Biosystems) for virtual slides. The measurements were made sequentially, each set after another (respectively HES slides, digital HES slides, IHC slides and digital IHC slides), blindfolded to the data obtained at the previous steps to minimize learning bias.

The potential impact of these factors on therapeutic decisions was measured. Each case was classified as low or high risk according to JSCCR criteria to estimate potential differences in therapeutic decisions arising from differences between observers and methods. The risk was defined as high, for T1 cancers, if at least one of the five following criteria were met: (i) a positive vertical margin (R1, automatically considered when piecemeal resection), (ii) SMI depth > 1000 μm, (iii) adenocarcinoma with poor differentiation including signet ring cell and mucinous carcinomas, (iv) grade 2–3 tumour budding, (v) presence of lymphovascular invasion (LVI). The results were also interpreted in terms of indications for surgery based on expected unpublished European guidelines, namely (i) a positive vertical margin (R1, automatically considered when piecemeal resection), (ii) adenocarcinoma with poor differentiation, (iii) presence of venous and/or lymphatic emboli, (iv) presence of high-grade budding and (v) SM invasion depth > 2000 μm.

### Statistical analysis

The level of significance was set at *p* < 0.05. To quantify inter and intra-observer reproducibility (IOR and IAR) for qualitative data the Fleiss-kappa statistic test was used. For quantitative data intra-class correlation coefficient was used. The values of kappa strength agreements were interpreted according to McHugh et al. [25]. The value of intra-class correlations (ICC) was according to Koo et al. [26]. All statistical analyses were done with R (version 4.0.3).

## Results

### Study population

A total of 98 patients were included (56.1% of male; median age of 71 years old): 98 samples (one sample by patient) were studied, 65 of which (66.3%) were endoscopic submucosal dissection, 22 (22.5%) endoscopic mucosal resection specimens and 11 (11.2%) endoscopic piecemeal mucosal resection specimens. Three samples were excluded because the infiltrating cells were no longer visible after IHC staining. The lesions ranged in size from 6 to 100 mm with an average of 37 mm and a median of 30 mm (Table 1). No significant difference was observed between the groups with and without piece meal resection except for the median follow-up time that is increased in the piece meal resection group (Table 1). This result is not surprising as the surveillance has to be more intense for these patients for which no information about the quality of resection is available.

**Table 1:** Clinical and main pathological characteristics of patients

	Total population	Population without piecemeal	Piecemeal population
	*n* = 98	*n* = 87	
Male, *n* (%)	54 (56.1%)	45 (51.7%)	9 (81.8%)
Median age in years [range]	71.3 [13.22]	72.2 [13.18]	66.00 [13.9]
Tumour localization, *n* (%)			
Right colon	21 (21.4%)	17 (19.5%)	4 (36.3%)
Transverse colon	9 (9.2%)	7 (8.1%)	2 (18.2%)
Left and sigmoid colon	36 (36.7%)	34 (39.1%)	2 (18.2%)
Rectum	32 (32.7%)	29 (33.3%)	3 (27.3%)
Type of endoscopic resection, *n* (%)			
Endoscopic submucosal dissection	65 (66.3%)	65 (74.7%)	
Mucosectomy resection	22 (22.5%)	22 (25.3%)	
Piecemeal mucosectomy resection	11 (11.2%)	-	11 (100%)
Median size in mm [± SD]	30 [24.5]	30 [24.7]	35 [27.13]
Invasion depth according to Ueno, *n* (%)			
< 1000 μm	17 (17.3%)	14 (16.1%)	3 (27.3%)
1000–2000 μm	20 (20.4%)	18 (20.7%)	2 (18.2%)
> 2000 μm	61 (62.2%)	57 (65.5%)	6 (54.5%)
Involved margin resection (R1), *n* (%)	24 (24.5%)	13 (14.9%)	NA
Pathological features, *n* (%)			
Poor differentiation	7 (7.1%)	6 (6.9%)	1 (9.1%)
Poorly differentiated cluster	11 (11.2%)	10 (11.5%)	1 (9.1%)
Signet ring contingent	0 (0%)	0 (0%)	0 (0%)
Significative budding (grade 2 and 3)	2 (2.0%)	1 (1.1%)	1 (9.1%)
Lymphatic invasion	6 (6.1%)	5 (5.7%)	1 (9.1%)
Venous invasion	3 (3.1%)	3 (3.4%)	0 (0%)
Indication for surgery according to JSCCR or European guidelines, *n* (%)	90 (91.8%)	79 (90.8%)	11 (100%)
Indication of surgery proposed by dedicated MDT, *n* (%)	53 (54.1%)	46 (52.9%)	7 (63.6%)
Surgery finally performed, *n* (%)	49 (50.0%)	43 (49.4%)	6 (54.5%)
Persistence of local tumour on surgical specimen, *n* (%)	3 (3.1%)	2 (2.3%)	1 (9.1%)
Lymph nodes involvement on surgical specimen, *n* (%)	3 (3.1%)	3 (3.5%)	0 (0%)
Vascular invasion on surgical specimen, *n* (%)	1 (1.0%)	1(1.5%)	0 (0%)
Median follow-up in months [± SD]	27.74 [23]	27.21 [20.7]	47.67 [32.30]
Recurrence, *n* (%)			
Local only	0	0	
Distant only	1 (1.0%)	1 (1.5%)	
Both	0	0	
Median recurrence-free survival in months [± SD]	30.8 [26.7]	29.8 [23.9]	42.9 [35.3]
Median overall survival in months [± SD]	30.8 [27.4]	30 [24.6]	47.67 [32.30]

*MDT*, multidisciplinary tumour board; *JSCCR*, Japanese Society for Cancer of the Colon and Rectum

Comment: All cases with which there was an interobserver discordance for a pejorative factor were reviewed between the three observers with physical slides and HES staining to obtain a consensus. For emboli, a complementary immunohistochemical study was performed, using CD-34 and D2-40 (podoplanin) antibody when there was still a doubt. An average of the infiltration depths was performed from the HES data under the microscope. A consensual surgical indication for surgery according to JSCCR guidelines was proposed

At the time of our study in December 2021, 1 (1%) patient presented with a recurrence of a dysplastic lesion without an infiltrating lesion 4 years after piecemeal resection. A total of 49 (50%) patients underwent subsequent colorectal surgery with lymphadenectomy and 3 (3.1%) of them had regional nodal metastases. Distant metastases were observed in 1 (1%) patient, without any CRC-related death. The patient’s clinical and pathological characteristics are presented in the Table 1.

### Distribution of pejorative histopronostic factors

In our study, most cases had an infiltration depth > 1000 μm according to the JSCCR method. Only one case had infiltration < 1000 μm and was associated with other pejorative histopronostic factors (Supplemental table 3). The patient in question did not have lymph node metastasis. Significant budding was found in 2 cases (2.0%), lymphatic invasion in 6 cases (6.1%) and veinous invasion in 3 cases (3.1%) (Table 1). The other aggressive pathological features linked to differentiation were more often found with a respective frequency of 7.1% for poor differentiation (7 cases) and 11.2% for poorly differentiated clusters (11 cases). It has to be noticed that no signet ring cell contingent was found.

### Reproducibility of infiltration’s depth

The Ueno and JSCCR methods had excellent interobserver reproducibility (IOR), with intra-class correlation coefficients (ICCs) of 0.858 and 0.903, respectively, on HES, under microscope. IHC analysis improved it further (ICC = 0.923 and 0.925) (Table 2). The JSCCR method obtained the best IAR between modalities (ICCs ranging from 0.738 to 0.894), except for the junior pathologist’s analysis on the digital slides (Table 3). The IAR between methods was poor (was poor to good) (Table 4).

**Table 2:** Summary of interobserver agreement with intra-class correlation coefficients (95% confidence intervals) between three raters for measuring the depth of infiltration according to the method and modality of observation

	GP vs VH	GP vs TF	VH vs TF	GP vs VH vs TF
Ueno				
Microscope HES	0.599 (0.427 to 0.718)	0.656 (0.548 to 0.742)	0.74 (0.647 to 0.81)	0.858 (0.804 to 0.897)
Microscope IHC	0.844 (0.789 to 0.886)	0.787 (0.714 to 0.842)	0.765 (0.687 to 0.826)	0.923 (0.898 to 0.943)
Digitized slide HES	0.303 (0.116 to 0.462)	0.236 (0.054 to 0.398)	0.691 (0.593 to 0.768)	0.696 (0.562 to 0.786)
Digitized slide IHC	0.818 (0.747 to 0.869)	0.797 (0.727 to 0.85)	0.809 (0.743 to 0.86)	0.927 (0.903 to 0.946)
Kitajima				
Microscope HES	0.17 (0.016 to 0.318)	0.326 (0.123 to 0.491)	0.559 (0.429 to 0.665)	0.651 (0.522 to 0.747)
Microscope IHC	0.643 (0.525 to 0.735)	0.388 (0.196 to 0.542)	0.535 (0.395 to 0.648)	0.757 (0.662 to 0.826)
Digitized slide HES	0.224 (0.063 to 0.374)	0.119 (− 0.025 to 0.264)	0.63 (0.44 to 0.749)	0.614 (0.416 to 0.738)
Digitized slide IHC	0.635 (0.52 to 0.727)	0.423 (0.269 to 0.554)	0.503 (0.368 to 0.617)	0.767 (0.69 to 0.827)
JSCCR				
Microscope HES	0.705 (0.611 to 0.779)	0.748 (0.658 to 0.816)	0.802 (0.733 to 0.854)	0.903 (0.871 to 0.928)
Microscope IHC	0.756 (0.676 to 0.819)	0.843 (0.787 to 0.885)	0.817 (0.753 to 0.865)	0.925 (0.901 to 0.945)
Digitized slide HES	0.47 (0.331 to 0.589)	0.479 (0.342 to 0.597)	0.915 (0.88 to 0.94)	0.83 (0.775 to 0.874)
Digitized slide IHC	0.836 (0.762 to 0.885)	0.839 (0.782 to 0.882)	0.853 (0.798 to 0.894)	0.942 (0.922 to 0.957)

*Vs*, versus; *HES*, haematoxylin-eosin-saffron; *IHC*, immunohistochemistry

**Table 3:** Summary of intra-observer agreement with intra-class coefficients (95% confidence intervals) between modalities for measuring the depth of infiltration according to methods of observation from three raters

	Microscope HES vs digitized slide HES	Microscope HES vs microscope IHC	Digitized slide HES vs digitized slide IHC	Microscope IHC vs digitized slide IHC
**Ueno**				
GP	0.607 (0.462–0.714)	0.645 (0.507–0.744)	0.435 (0.195–0.603)	0.906 (0.87–0.932)
VH	0.802 (0.733–0.854)	0.766 (0.688–0.827)	0.648 (0.542–0.735)	0.78 (0.705–0.837)
TF	0.597 (0.479–0.693)	0.629 (0.518–0.719)	0.638 (0.529–0.727)	0.793 (0.723–0.847)
**Kitajima**				
GP	0.415 (0.269–0.542)	0.52 (0.364–0.642)	0.214 (0.053–0.364)	0.688 (0.59–0.766)
VH	0.597 (0.48–0.694)	0.523 (0.392–0.634)	0.465 (0.326–0.585)	0.612 (0.497–0.706)
TF	0.717 (0.626–0.789)	0.804 (0.737–0.856)	0.75 (0.668–0.814)	0.749 (0.665–0.814)
**JSCCR**				
GP	0.441 (0.298–0.565)	0.738 (0.652–0.805)	0.461 (0.32–0.582)	0.911 (0.877–0.936)
VH	0.857 (0.805–0.895)	0.765 (0.687–0.826)	0.894 (0.856–0.923)	0.86 (0.809–0.897)
TF	0.852 (0.794–0.893)	0.88 (0.836–0.913)	0.871 (0.825–0.906)	0.83 (0.769–0.876)

*Vs*, versus; *HES*, haematoxylin-eosin-saffron; *IHC*, immunohistochemistry

**Table 4:** Summary of intra-observer agreement with intra-class coefficients (95% confidence intervals) between methods for measuring the depth of invasion according to the observation modality from three raters

	Ueno vs Kitajima	Ueno vs JSCCR	Kitajima vs JSCCR
Microscope HES			
GP	0.403 (0.249 to 0.536)	0.607 (0.367 to 0.746)	0.24 (0.028 to 0.42)
VH	0.4 (0.25 to 0.531)	0.938 (0.903 to 0.959)	0.392 (0.223 to 0.533)
TF	0.294 (0.136 to 0.438)	0.641 (0.438 to 0.763)	0.664 (0.539 to 0.756)
Microscope IHC			
GP	0.372 (0.217 to 0.507	0.818 (0.676 to 0.888	0.255 (0.069 to 0.418)
VH	0.535 (0.404 to 0.643)	0.755 (0.657 to 0.824)	0.385 (0.216 to 0.526)
TF	0.276 (0.118 to 0.421)	0.671 (0.481 to 0.784)	0.624 (0.507 to 0.718)
Digitized slide HES			
GP	0.378 (0.229 to 0.51)	0.118 (− 0.022 to 0.26)	0.053 (− 0.062 to 0.177)
VH	0.538 (0.405 to 0.648)	0.778 (0.596 to 0.865)	0.38 (0.169 to 0.543)
TF	0.814 (0.75 to 0.863)	0.884 (0.812 to 0.925)	0.759 (0.677 to 0.822)
Digitized slide IHC			
GP	0.603 (0.48 to 0.701)	0.767 (0.607 to 0.852)	0.405 (0.192 to 0.566)
VH	0.721 (0.628 to 0.792)	0.767 (0.644 to 0.843)	0.50 (0.321 to 0.646)
TF	0.413 (0.267 to 0.542)	0.808 (0.688 to 0.876)	0.544 (0.411 to 0.653)

*Vs*, versus; *HES*, haematoxylin-eosin-saffron; *IHC*, immunohistochemistry

### Reproducibility of other prognostic factors

Regarding poorly differentiated clusters, lymphatic invasion and venous invasion on microscope examination of HES, IOR was poor (*κ* = 0.141, 0.196 and 0.313, respectively) (Table 5). For budding, the IOR of microscope evaluation was poor, either with the recommended classification [5] or when classified as non-significant/significant (i.e. no budding or grade 1 versus grade 2 or 3) (*κ*= 0.122 and 0.172, respectively). Microscope IHC analysis seemed to be better, reaching moderate IOR (*κ* = 0.560 for the three-tiered classification and 0.714 for binary classification). More high-grade and significant budding cases were detected. Digital analysis did as well as microscope examination (Table 5).

**Table 5:** Summary of interobserver agreement measurement by Fleiss’s kappa coefficient for qualitative and semi-qualitative data, from three raters

	Results	GP	VH	TF	Kappa	Global kappa (quality of agreement)
Polyp form	Pedunculated	20 (20.2%)	19 (19.19%)	17 (17.17%)	0.67	0.67 (moderate)
	Sessile	79 (79.8%)	80 (80.81%)	82 (82.83%)		
Condition of the *muscularis mucosae*	A	11 (11.11%)	21 (21.21%)	22 (22.22%)	0.525	0.499 (poor agreement)
	B	37 (37.37%)	12 (12.12%)	25 (25.25%)	0.370	
	C	51 (51.52%)	66 (66.66%)	52 (52.53%)	0.602	
Vertical margin*	R0	83 (84.69%)	91 (92.86%)	81 (82.65%)	0.586	0.586 (poor agreement)
	R1	15 (15.31%)	7 (7.14%)	17 (17.35%)		
Tumour differentiation (WHO 2019)	Low grade	93 (93.94%)	92 (92.93%)	90 (90.91%)	0.313	0.313 (minimal agreement)
	High grade	6 (6.06%)	7 (7.07%)	9 (9.09%)		
Tumour differentiation (WHO 2010)	Well differentiated	24 (24.24%)	14 (14.14%)	40 (40.4%)	0.374	0.326 (minimal agreement)
	Moderately differentiated	69 (69.7%)	78 (78.79%)	50 (50.51%)	0.291	
	Poorly differentiated	6 (6.06)	7 (7.07)	9 (9.09)	0.313	
Signet ring contingent	Absent	98 (98.99%)	97 (97.98%)	97 (97.98%)	0.186	0.186 (no agreement)
	Present	1 (1.01%)	2 (2.02%)	2 (2.02%)		
Mucinous contingent	Absent	71 (71.72%)	76 (76.77%)	74 (74.75%)	0.788	0.788 (moderate agreement)
	Present	28 (28.28%)	23 (23.23%)	25 (25.25%)		
Poorly differentiated cluster	Absent	79 (79.8%)	88 (88.89%)	75 (75.76%)	0.42	0.42 (poor agreement)
	Present	20 (20.2%)	11 (11.11%)	24 (24.24%)		
Lymphatic invasion	Absent	97 (97.98%)	94 (94.95%)	90 (90.91%)	0.141	0.141 (no agreement)
	Present	2 (2.02%)	5 (5.05%)	9 (9.09%)		
Venous invasion	Absent	93 (93.94%)	94 (94.95%)	90 (90.91%)	0.196	0.196 (no agreement)
	Present	6 (6.06%)	5 (5.05%)	9 (9.09%)		
Microscope HES tumour budding	Grade 1 (0–4 buds)	94 (94.95%)	99 (100%)	86 (86.87%)	0.172	0.122 (no agreement)
	Grade 2 (5–9 buds)	4 (4.04%)	0 (0%)	8 (8.08%)	0.045	
	Grade 3 (≥ 10 buds)	1 (1.01%)	0 (0%)	5 (5.05%)	0.149	
	Not significant (grade 1)	94 (94.95%)	99 (100%)	86 (86.87%)	0.172	0.172 (no agreement)
	Significant (grade 2 and 3)	5 (5.05%)	0 (0%)	13 (13.13%)		
Microscope IHC tumour budding	Grade 1 (0–4 buds)	67 (67.68%)	58 (58.59%)	59 (59.6%)	0.714	0.560 (poor agreement)
	Grade 2 (5–9 buds)	14 (14.14%)	26 (26.26%)	21 (21.21%)	0.340	
	Grade 3 (≥ 10 buds)	18 (18.18%)	15 (15.15%)	19 (19.19%)	0.627	
	Not significant (grade 1)	67 (67.68%)	58 (58.59%)	59 (59.6%)	0.714	0.714 (moderate agreement)
	Significant (grade 2 and 3)	32 (32.32%)	41 (41.41%)	40 (40.4%)		
Digitized HES tumour budding	Grade 1 (0–4 buds)	92 (92.93%)	72 (72.73%)	83 (83.84%)	0.254	0.249 (minimal agreement)
	Grade 2 (5–9 buds)	6 (6.06%)	19 (19.19%)	10 (10.1%)	0.126	
	Grade 3 (≥ 10 buds)	1 (1.01%)	8 (8.08%)	6 (6.06%)	0.368	
	Not significant (grade 1)	92 (92.93%)	72 (72.73%)	83 (83.84%)	0.254	0.254 (minimal agreement)
	Significant (grade 2 and 3)	7 (7.07%)	27 (27.27%)	16 (16.16%)		
Digitized IHC tumour budding	Grade 1 (0–4 buds)	60 (60.61%)	42 (42.42%)	57 (57.58%)	0.675	0.538 (poor agreement)
	Grade 2 (5–9 buds)	11 (11.11%)	23 (23.23%)	16 (16.16%)	0.230	
	Grade 3 (≥ 10 buds)	28 (28.28%)	34 (34.34%)	26 (26.26%)	0.709	
	Not significant (grade 1)	60 (60.61%)	42 (42.42%)	57 (57.58%)	0.675	0.675 (moderate agreement)
	Significant (grade 2 and 3)	39 (39.39%)	57 (57.58%)	42 (42.42%)		

Condition of muscularis mucosae: A, clearly identified; B, incompletely disrupted with deformity; C, completely disrupted; vertical margin status: *R0*, 0 mm margin; *WHO*, World Health Organization; *HES*, haematoxylin-eosin-saffron; *IHC*, immunochemistry; *one case was not applicable because of a completely tangential inclusion

### Additional surgery

Regarding theoretical indications for additional surgery according to JSCCR recommendations (Table 6) IOR based on microscopic HES analysis was moderate (*κ* = 0.607). IHC analysis improved it (*κ* = 0.763). Digital pathology analysis was even more reproducible when combined with IHC analysis (*κ* = 0.802).

**Table 6:** Summary of interobserver agreement by Fleiss’s kappa coefficient on the surgical indication according to the JSCCR criteria and to the different modalities

	Surgery indication	GP	VH	TF	Kappa (quality of agreement)
Microscope HES	Yes	95 (95.96%)	88 (88.89%)	89 (89.9%)	0.607 (moderate agreement)
	No	4 (4.04%)	11 (11.11%)	10 (10.1%)	
Microscope IHC	Yes	94 (94.95%)	92 (92.93%)	93 (93.94%)	0.763 (moderate agreement)
	No	5 (5.05%)	7 (7.07%)	6 (6.06%)	
Digitized HES	Yes	94 (94.95%)	91 (91.92%)	92 (92.93%)	0.625 (moderate agreement)
	No	5 (5.05%)	8 (8.08%)	7 (7.07%)	
Digitized IHC	Yes	94 (94.95%)	94 (94.95%)	93 (93.94%)	0.802 (strong agreement)
	No	5 (5.05%)	5 (5.05%)	6 (6.06%)	

*HES*, haematoxylin-eosin-saffron; *IHC*, immunohistochemistry

IOR for surgery indications based on forthcoming European recommendations was increased with IHC (*κ* = 0.659) (Table 7). Furthermore, the number of cases in which surgery would have been indicated was not significantly different between HES and IHC analysis for the two recommendations (Tables 6 and 7). Digital pathology did not change significantly the IOR.

**Table 7:** Summary of interobserver agreement by Fleiss’s kappa coefficient on the surgical indication according to likely future European recommendations to be published and to the different modalities

	Surgery indication	GP	VH	TF	Kappa (quality of agreement)
Microscope HES	Yes	85 (85.85%)	79 (79.8%)	86 (86.87%)	0.52 (poor agreement)
	No	14 (14.14%)	20 (20.20%)	13 (13.13%)	
Microscope IHC	Yes	89 (89.9%)	86 (86.87%)	89 (89.89%)	0.659 (moderate agreement)
	No	10 (10.1%)	13 (13.13%)	10 (10.1%)	
Digitized HES	Yes	82 (82.83%)	87 (87.88%)	87 (87.88%)	0.604 (moderate agreement)
	No	17 (17.17%)	12 (12.12%)	12 (12.12%)	
Digitized IHC	Yes	87 (87.88%)	91 (91.91%)	93 (93.93%)	0.621 (moderate agreement)
	No	12 (12.12%)	8 (8.08%)	6 (6.06%)	

*HES*, haematoxylin-eosin-saffron; *IHC*, immunohistochemistry

Among the 90 theoretical indications of additional surgery, 53 were proposed during the dedicated MDT and 49 patients underwent this surgery: persistent local tumour was found in 3 patients and 3 other had lymph nodes involvement. The 3 patients with local recurrence presented deep infiltration > 2000 for 2 of them no matter the method used to establish that measure, whereas the third had a deep invasion measure that was varying between > 1000 or > 2000 depending on the method. Three patients with lymph node metastasis all presented one aggressive feature. The first presented veinous invasion, the second poor differentiation and the third a deep invasion > 2000 μm. After a median follow-up of 27.7 months, the median recurrence-free survival was 30.8 months (Table 1).

## Discussion

This study was carried out on endoscopic resection specimens only, on the contrary to most of the other studies in the literature. These are biased by selection towards more severe endoscopic patterns, for which surgery was indicated in the first place [7, 9, 27]. The limitations of our study include learning effect from the sequentially analyzed cases. Besides, it may be relevant to consider pedunculated and sessile polyps separately, as that the risk of metastasis is lower for the former and the SMI is probably a more important factor for the latter [7, 28–30]. However, in our study, there was no differences between the two groups (Table 1).

**Fig. 2:**
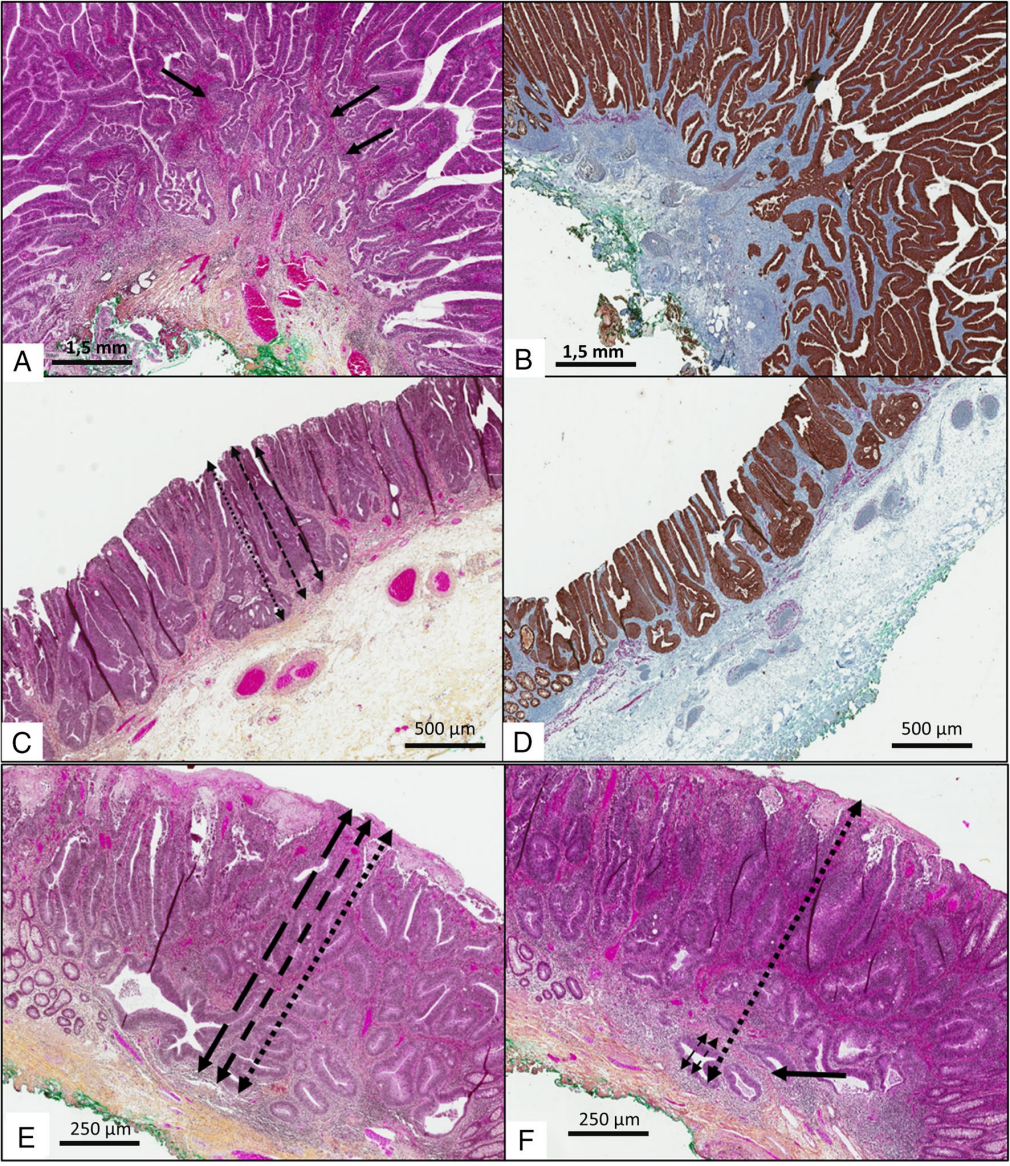
Difficulties for submucosal infiltration depth measurement in **A**, **C**, **D**, **E** HES- and **B** and **D** IHC-stained sections (100 × magnification) of colorectal adenocarcinoma in a sessile polyp specimen. In this first case, **A** HES staining suggests that *muscularis mucosae* fibres are present (arrows) while **B** IHC staining reveals that there is none. In the second case, note how a very small lesion with estimable muscularis mucosae in HES (**C**) but destroyed in IHC (**D**) can exceed the depth threshold of 1000 μm (here, 1162 μm) if measured using the JSCCR (double-headed black round dot arrow), Ueno (double-headed black dashes arrow) and Kitajima (double-headed long dashes arrow) methods. In the third case, depending on the block selected, the muscularis mucosae appears completely ruptured (**E**) or assessable (**F**) (black single arrow). The invasion depth is 328 μm if measured using the Ueno and Kitajima method (double-headed black dashes arrow and double-headed long black dashes arrow respectively) and 2459 μm if measured using the JSCCR method (double-headed black round spot arrow)

**Fig. 3:**
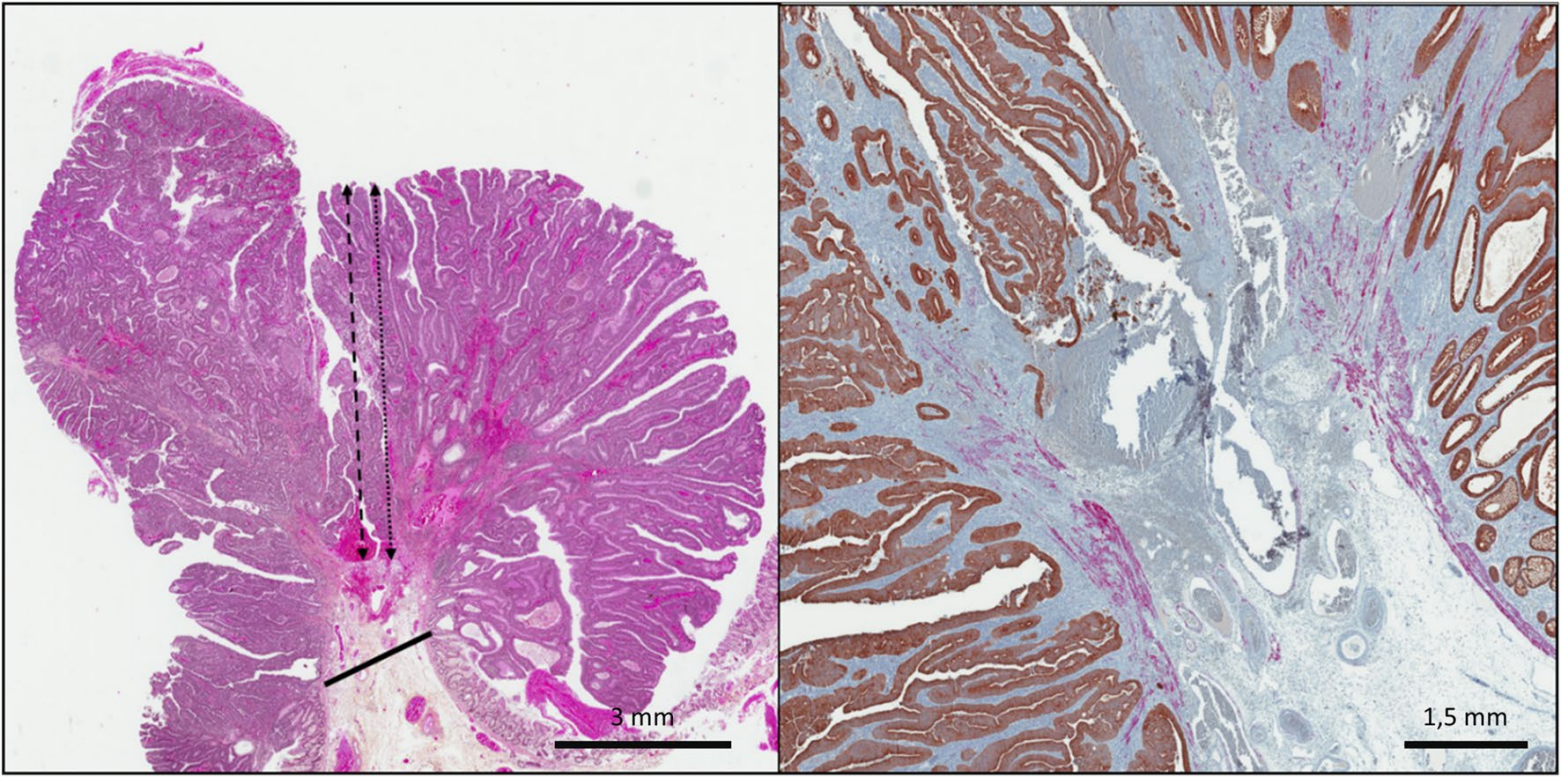
Difficulties for submucosal invasion depth measurement in **A** HES- and **B** IHC-stained sections (100 × magnification) of colorectal adenocarcinoma in a pediculated polyp specimen. Example of a major discrepancy between measurement methods for a pedunculated polyp. The black line represents level 2 of the Haggit classification. The invasion does not extend below this line (**A**). According to Kitajima et al., this is a case of « head invasion ». The double-headed black round dot and the double-headed black dashes arrows show the depth of invasion (6.7 mm) measured from the surface of the lesion (as in the JSCCR and Ueno methods respectively) in cases where the muscularis mucosae is destroyed (**B**)

The depth of submucosal invasion is one of the key factors for additional surgery decision. However, there is still no consensus about the measurement method and the staining to use to obtain a robust criterion (Fig. 2 and 3).

To our knowledge, this is the first study to have evaluated IOR between observers with different experiences both using three different methods (Ueno, Kitajima and JSCCR) and different histological technics including IHC and digital pathology. The fact that Ueno and JSCCR methods had excellent IOR, particularly on IHC analysis should be considered for future recommendations. The use of digital pathology was equivalent and did not reduce IOR. The good IOR of the Ueno method was consistent with other reports by several authors with ICCs varying from 0.89 in Barel et al. study to [14] 0.64 in Wang et al.’s work [13]. The JSCCR method IAR was excellent, except for those of the least experienced observer, whereas the Ueno method IAR was not affected by the experience of the pathologist. This may be linked to the complexity of the JSCCR method compared to Ueno’s. However, with the Ueno method, agreement between HES and IHC results was lower. This is explained by the ability with IHC staining to better identify the MM fibres and thus adjust the upper level of the SM layer (Fig. 2). Digital pathology also seems to be impacted by the experience of the pathologist as the IAR (microscope versus digitalized HES) was moderate to good except for the junior pathologist.

IAR is highly variable when comparing one measurement method to another. These results may explain why different measurement thresholds have been established in different studies, ranging from 1000 to 3000 μm. Indeed, the daily practice of these methods do not give concordant measurements. Therefore, we recommend that future recommendations mention to always report which measurement method was used [7, 9, 31].

Both Ueno and Kitajima methods, are based on subjective evaluation of MM integrity. As in our study, Davenport et al. and Kitajima et al. found it hard to evaluate the MM status. While not perfect, IHC can resolve certain ambiguities. The JSCCR method is much stricter in that SMI depth is measurement, although it is important to bear in mind that the aspect of the MM can differ a lot between sections (Fig. 3). The JSCCR method is therefore highly reproducible at the cost of SMI depth overestimation. Supporting this statement, Kouyama et al. and Yoshida et al. reported that depth measurements they made from the surface of the lesion were in all cases > 1000 μm [32, 33] leading to many surgeries.

Regarding the IOR of other prognostic factors, which lead to complementary surgery on their one, the rarity of these events makes the *κ* difficult to interpret, as in other studies of sCRC endoscopic treatment. However, the proportions of cases in which these features were observed were consistent between techniques and similar to those reported in the literature for poor differentiation and signet ring cells [7, 8, 14, 34]. LVI was found between 8 and 14.6% of cases, when we found 2–9% of cases for lymphatic emboli and 6–9% of cases for venous emboli [14, 9, 15]. The distinction between lymphatic and veinous emboli may also be relevant as it is not linked to the same pathological mechanism. However, the percentage of tumours with positive vertical margins was lower in the present study (7 to 15%) than in Barel et al.’s (38%) [14]. We believe this is because of our study’s setting in a tertiary endoscopy centre with high resection volume. Although the IOR of each histopronostic criteria considered here was low, indications for surgery based on multiple factors agreed much better.

Regarding budding, as *κ* is highly dependent on the number of observed events, the low *κ* on HES mainly reflects that there were few cases with grade 2/3 budding. Our results show that IHC might improve reproducibility with a three-tiered system (grade 1 and grade 3 being the most reproducible). However, the highest IOR (*κ* = 0.714) was achieved by using a two-tiered system (significant or not) with IHC. As two-tiered classifications are more reproducible, this should be considered for future recommendations. Since many more buds were detected with IHC than with HES staining, it should be kept in mind that it may have a direct impact on patient management. That’s why to this day, IHC is not recommended in guidelines so far. To be recommended, IHC needs to be more studied to define specific thresholds adapted to the fact that more buds are counted with this technic. Even with IHC, the IOR only achieve moderate agreement. Indeed, there are many pitfalls in the evaluation of budding [12, 16]. Previous studies are consistent on the role of IHC for budding detection, with an even stronger impact in the Barel et al. study (HES *κ* = 0.235 versus IHC *κ* = 0.842 [14, 17].

In terms of theoretical indications for surgery according to JSCCR recommendations or forthcoming European ones, moderate reproducibility (*κ* = 0.607 to 0.763) is explained in part by the low prevalence of cases with no indication for surgery. The fact that nearly all cases had an indication for surgery is mostly explained by the measurement method, which increases the likelihood of measurements > 1000 μm [12, 32, 35].

UK recommendations mentioned that strict application of the JSCCR recommendations leads to overuse of surgery [35]. In our practice, after considering the patients’ comorbidities and their wishes, the number of patients who underwent surgery is much smaller (*n* = 53 (54%)) (Table 1). A posteriori, in 41% of cases, the therapeutic management did not follow the recommendations of the JSCCR. However, it does not seem to impact the patient prognosis, as with close follow-up, although less than 5 years median, only one patient developed distant metastasis without death from colorectal cancer occurring.

Importantly, for the first time, a study shows that digital pathology achieves the same levels of reproducibility as microscope on all factors studied. This is an important condition for its use, which will probably become more and more widespread in the coming years. The main point with digital pathology is to improve and accelerate consultation between pathologists from several centres to respond more accurately and quickly to patient management problems.

## Conclusions

In conclusion, although most histopronostic factors associated with the occurrence of lymph node metastases have poor measurement reproducibility, here and in the literature, our results suggest that their combined use in therapeutic decision making compensates for the variability of each factor and yields clinically acceptable levels of reproducibility. This study also indicates that IHC facilitates the evaluation of certain criteria and may therefore improve the reproducibility of these assessments. Digital analyses could be used as the reproducibility is like microscope examination. Finally, we call for new recommendations or consensus for daily practice pathological assessment of endoscopic specimens, as there is still a lot of specific issues that remain unclarified and to raise the question about the relevance of the threshold to 1000 μm.

### Supplementary information

ESM 1 (DOCX 22 kb)

## Data Availability

The datasets generated during and/or analyzed during the current study are available from the corresponding author on reasonable request.
