# Supplementary material for: Histopronostic factors in superficial colorectal adenocarcinomas treated by endoscopy: reproducibility and impact of immunohistochemistry and digital pathology
Source: Virchows Arch. 2024 Jan 26;485(2):233–44. doi: 10.1007/s00428-023-03722-3 (PMC11329611; doi:10.1007/s00428-023-03722-3)
Supplement: Supplementary file 1 — (DOCX 22 kb) [file 428_2023_3722_MOESM1_ESM.docx]

Supplemental Tables

**Supplemental table 1: summary of interobserver agreement by Fleiss's Kappa coefficient on the surgical indication according to likely future European recommendations to be published without infiltration depth threshold and to the different modalities.**

|  | Surgery Indication | GP | VH | TF | Kappa (quality of agreement) |
| --- | --- | --- | --- | --- | --- |
| Microscope HES | Yes | 34 (34.34%) | 28 (28.28%) | 46 (46.46%) | 0.433  (poor agreement) |
|  | No | 65 (65.66%) | 71 (71.72%) | 53 (53.54%) |  |
| Microscope IHC | Yes | 51 (51.52%) | 59 (59.6%) | 64 (64.65%) | 0.514  (poor agreement) |
|  | No | 48 (48.48%) | 40 (40.4%) | 35 (35.35%) |  |
| Digitized HES | Yes | 34 (34.34%) | 48 (48.48%) | 49 (49.49%) | 0.358  (minimal agreement) |
|  | No | 65 (65.66%) | 51 (51.52%) | 50 (50.51%) |  |
| Digitized IHC | Yes | 56 (56.57%) | 70 (70.71%) | 65 (65.66%) | 0.504  (poor agreement) |
|  | No | 12 (12.12%) | 8 (8.08%) | 6 (6.06%) |  |
| HES = haematoxylin-eosin-saffron ; IHC = immunohistochemistry. | | | | | |

**Supplemental table 2: summary of the intra-observer agreement measurement by Fleiss’s Kappa coefficient between modalities for evaluating budding from three raters.**

|  |  | **Microscope HES versus digitized slide HES** | **Microscope HES versus microscope IHC** | **Digitized slide HES versus digitized slide IHC** | **Microscope IHC versus digitized slide IHC** |
| --- | --- | --- | --- | --- | --- |
| **GP** | Grade 1 | 0.823 | 0.036 | 0.037 | 0.846 |
|  | Grade 2 | 0.789 | 0.144 | -0.094 | 0.222 |
|  | Grade 3 | 1.000 | -0.106 | -0.172 | 0.660 |
|  | Non significant | 0.823 | 0.036 | 0.037 | 0.846 |
|  | Significant |  |  |  |  |
| **VH** | Grade 1 | -0.158 | -0.261 | 0.214 | 0.596 |
|  | Grade 2 | -0.106 | -0.151 | -0.027 | 0.105 |
|  | Grade 3 | -0.042 | -0.082 | 0.154 | 0.485 |
|  | Non significant | -0.158 | -0.261 | 0.214 | 0.596 |
|  | Significant |  |  |  |  |
| **TF** | Grade 1 | 0.556 | 0.150 | 0.122 | 0.750 |
|  | Grade 2 | 0.267 | 0.071 | -0.063 | 0.236 |
|  | Grade 3 | 0.711 | 0.052 | 0.105 | 0.626 |
|  | Non significant | 0.556 | 0.15 | 0.122 | 0.75 |
|  | Significant |  |  |  |  |
| Vs : versus ; HES = haematoxylin-eosin-saffron ; IHC = immunohistochemistry. | | | | | |

**Supplemental table 3: Distribution of histopronostic criteria, based on consensus between three observers, according to the depth of infiltration according to JSCCCR, Ueno or Kitajima recommandations.**

| **Depth invasion** | **Number of cases whitout piecemal [number of piecemeal resection cases]** | **High grade adenocarcinoma** | **Poorly differenciated cluster** | **Significative Budding** | **Lymphatic invasion** | **Veinous invasion** | **Signet ring contingent** |  |
| --- | --- | --- | --- | --- | --- | --- | --- | --- |
| Based on JSCCCR recommendations | | | | | | | |  |
| **<1000 µm** | **9 [3]** | 1* | 1* | 0 | 1* | 0 | 0 |  |
|  |  |  |  |  |  |  |  |  |
| **1000-2000 µm** | **12 [3]** | 0 | 1 | 0 | 2 (1) | 0 | 0 |  |
|  |  |  |  |  |  |  |  |  |
| **>2000 µm** | **66 [5]** | 5 (1) | 8 (1) | 1 (1) | 2 | 3 | 0 |  |
|  |  |  |  |  |  |  |  |  |
| Based on Ueno recommendations | | | | | | | |  |
| **<1000 µm** | **14 [3]** | 1* | 1* | 0 | 1* | 0 | 0 |  |
|  |  |  |  |  |  |  |  |  |
| **1000-2000 µm** | **18 [2]** | 1 | 1 | 0 | 2 (1) | 0 | 0 |  |
|  |  |  |  |  |  |  |  |  |
| **>2000 µm** | **41 [6]** | 4 (1) | 8 (1) | 1 (1) | 2 | 3 | 0 |  |
|  |  |  |  |  |  |  |  |  |
| Based on Kitajima recommendations | | | | | | | |  |
| **<1000 µm** | **18 [3]** | 2* | 2* | 0 | 2* | 0 | 0 |  |
|  |  |  |  |  |  |  |  |  |
| **1000-2000 µm** | **17 [5]** | 1 | 2 | 1 | 1 (1) | 0 | 0 |  |
|  |  |  |  |  |  |  |  |  |
| **>2000 µm** | **52 [3]** | 3 (1) | 6 (1) | -1 | 2 | 3 | 0 |  |
|  |  |  |  |  |  |  |  |  |

* The same case with SMI depth <1000 µm presented at the same time the three histopronostic criteria

Comment: All cases for which there was an interobserver discordance for a pejorative factor were reviewed between the three observers with physical slides and HES staining to obtain a consensus. For emboli, a complementary immunohistochemical study was performed, using CD-34 and D2-40 (podoplanin) antibody when there was still a doubt. An average of the infiltration depths was performed from the HES data under the microscope. A consensual surgical indication for surgery according to JSCCR guidelines was proposed.
